# Gene expression changes in *Porphyromonas gingivalis* W83 after inoculation in rat oral cavity

**DOI:** 10.1186/s12866-015-0438-0

**Published:** 2015-05-24

**Authors:** Jian Zhao, Qian Li, Chun-Ling Pan, Jun-Chao Liu, Hong-Yan Wang, Li-Si Tan, Ya-Ping Pan

**Affiliations:** Department of Periodontology, School of Stomatology, China Medical University, Shenyang , Liaoning China

**Keywords:** Porphyromonas gingivalis, Periodontitis, Microarray, Gene expression

## Abstract

**Background:**

The development of chronic periodontitis was due to not only periodontal pathogens, but also the interaction between periodontal pathogens and host. The aim of this study is to investigate the alterations in gene expression in *Porphyromonas gingivalis* (*P.gingivalis*) W83 after inoculation in rat oral cavity.

**Results:**

*P.gingivalis* W83 inoculation in rat oral cavity caused inflammatory responses in gingival tissues and destroyed host alveolar bone. Microarray analysis revealed that 42 genes were upregulated, and 22 genes were downregulated in the detected 1786 genes in the inoculated *P.gingivalis* W83. Real-time quantitative PCR detection confirmed the expression alterations in some selected genes. Products of these upregulated and downregulated genes are mainly related to transposon functions, cell transmembrane transportation, protein and nucleic acid metabolism, energy metabolism, cell division and bacterial pathogenicity.

**Conclusions:**

*P.gingivalis* W83 has a pathogenic effect on host oral cavity. Meanwhile, inflammatory oral environment alters *P.gingivalis* W83 gene expression profile. These changes in gene expression may limit the proliferation and weaken the pathogenicity of *P.gingivalis* W83, and favor themselves to adapt local environment for survival.

## Background

Periodontitis is a chronic inflammatory disorder mediated by host and bacteria interactions and manifested by damage to the periodontal tissues that may progress to tooth loss. The host inflammatory responses stimulated by periodontal pathogens intend to eliminate the invaded bacteria and attribute to the destruction of tooth supporting tissues and tooth loss [1]. Moreover, the local periodontal environment may change the gene expression profile of periodontal pathogens [2–4]. To a certain extent, the variation of bacterial gene expression may alter the pathogenic ability of bacteria.

*Porphyromonas gingivalis* (*P.gingivalis*) is an opportunistic pathogen of the oral mucosa and a prominent member of the oral biofilms. It is well known that *P.gingivalis* is implicated in the onset and progression of chronic periodontitis. *P.gingivalis* can induce immune cells to secrete cytokines when they invade into hosts. These cytokines are present in inflamed gingiva and aggravate the destruction of oral gingival tissues and alveolar bone [5]. In the meantime, the expression of *P.gingivalis* genes varies under different conditions, such as iron or hemin [6,7], polyphosphate [8], rhein [9]. *P.gingivalis* may up-regulate or downregulate gene expression to adapt environment and survive [10].

The development of chronic periodontitis was not only due to periodontal pathogens, but also the interaction between periodontal pathogens and host. Most researches focus on periodontal pathogens acting on hosts, but ignore the action of host on *P.gingivalis*. Actually, the changes in *P.gingivalis* gene expression may affect the progression of chronic periodontitis. In the present study, the differential gene expression in *P.gingivalis* W83 inoculated in rat oral cavity and wild strain was analyzed.

## Methods

### Ethical statement

All rats were manipulated in accordance with Animal Research Reporting In Vivo Experiments (ARRIVE) guidelines. The experimental protocols were approved by the ethical committee of China Medical University.

### Bacteria and animals

This study was carried out with 6-week-old SPF rats (180 − 220 g) provided by Department of Experimental Animals, China Medical University, and maintained in a temperature-controlled room (23 ± 1 °C). *P.gingivalis* W83 was obtained from the American Type Culture Collection (ATCC) and grown anaerobically (10 % CO_2_, 10 % H_2_, 80 % N_2_) in enriched brain-heart infusion (BHI) broth containing 5 % fiber-free sheep blood, 1 % vitamin K and hemin, at 37 °C.

### *P.gingivalis* W83 inoculation

12 Rats were given azithromycin (10 mg/500 ml) *ad libitum* for 4 days to reduce the original oral flora. This was followed by a 7-day antibiotic-free period. 6 Rats were then orally challenged with *P. gingivalis* W83 (1 × 10^9^ CFU) by gavage into the esophagus and oral cavity five times every other day [11]. The other 6 rats (control group) were only challenged with BHI broth. All 12 rats received steel wire ligature in cervical part in two sides of first molars and an 8-week high sugar feeding.

### Alveolar bone loss analysis

Horizontal bone loss was assessed morphometrically by measuring the distance between the cement − enamel junction and the alveolar bone crest of the first, the second and the third molar. The alveolar bone destruction was detected by morphological and macroscopic observation, radiographic (PLANMECA, Finland) and stereomicroscope (SZX12, Olympus, Japan) fitted with a DIGIMED Viewer imaging measurement system evaluation at 6 sites per molars. Alveolar bone loss of every molar was presented in the figures as mean ± SD. Independent samples *t*-test was used to calculate the significance among the groups (SPSS Inc., Chicago, IL, USA). *P*-value < 0.05 was considered statistically significant.

### Isolating culture and acquiring plaque

After *P.gingivalis* W83 inoculation in rat oral cavity for 8 weeks, plaques were acquired from periodontal pockets of first molar using toothpicks and put into 0.5 ml transfer tube. The plaques were dispersed by oscillator. 100 μl ten-fold serial dilutions were inoculated on BHI culture medium anaerobically at 37 °C for 5–7 days. The morphology of colonies was observed in primary cultures. *P.gingivalis*W83 colonies were identified by their black pigmentation, gram staining and PCR. The single clone was purified in BHI medium for subcultures in order to detect the differences in the gene of *P.gingivalis*W83.

### Microarray hybridization

3 samples were picked up from wild strain *P.gingivalis* W83 and inoculated *P.gingivalis* W83, respectively. The total RNA was extracted and labeled with Klenow, and then hybridism with *P.gingivalis* W83 chip. The commercial GeneChip *P.gingivalis* W83 Genome Array used here was provided by CapitalBio Corporation (http://www.capitalbio.com/, Beijing, China), a service provider authorized by Roche NimbleGen (Wisconsin, USA). Array hybridization, washing, scanning and data analysis were performed at the CapitalBio Corporation, Beijing, China and carried out according to the NimbleGen’s Expression user’s guide.

### Real-time quantitative PCR

To independently confirm the expression data generated by the microarray experiments, we performed real-time quantitative PCR analyses for 14 genes differentially regulated. Total RNA was extracted. Quality and concentration of the RNA were determined by measuring its absorbance at 260 and 280 nm using a microplate reader (M-200, Tecan, Switzerland). Total bacterial RNA was subsequently reverse-transcribed using the M-MLV RTase cDNA Synthesis Kit (Takara, China) following the manufacturer’s protocol. Real-time quantitative PCR analysis was conducted in an ABI Prism 7500 Sequence Detection System (Applied Biosystems, Foster City, CA, USA) in combination with the SYBR® Premix Ex TaqTM II PCR Master Mix Reagents Kit (Takara), as recommended by the manufacturer of the Wall Clear PCR Strip Tubes (Axygen, USA). The primers for the real-time quantitative PCR analysis were designed using Primer3 (http://bioinfo.ut.ee/primer3/) (Table 1). *P.gingivalis* W83 16 s DNA was used as the internal reference. Real-time quantitative PCR was performed three times for each sample. The data were analyzed according to relative gene expression by the 2-^ΔΔ^Ct method.

**Table 1:** Sequences for real-time PCR

Gene	Sequence(5’-3’)	PCR product (bp)
PG1005	F: CGGTGAGGTTTACAGAAGAA	79
	R: AGGGAGGTGTAAGTCACG	
PG1006	F: GGAATGGAGCGAAAGACC	169
	R: CCAACAAGCAGAACCGAC	
PG1007	F: TCTGTTTGTTTGTCCCATTC	62
	R: TATGGCTCCTCAAAGTAGAG	
PG1008	F: TTACAACAGCGGCTACCA	103
	R: TATCCACTGCCACAGCCT	
PG1009	F: AAGCGTGCTACCATTGCG	78
	R: TCAGGCTATACCCGTTCT	
PG1010	F: TCTGTCCCTGCGATACCT	99
	R: CACTCATCCTCCCTATCTTTC	
PG0874	F: AGGGTGTCTGAGCAAGTA	73
	R: TGGAGGAATCGAAGATAGAA	
PG1513	F: GAAACGGCTCAAGTCATA	114
	R: TCCCTCCTCCATTTCCAC	
PG0684	F: GAATACGGAGGTCAATCGC	90
	R: GAACGCTGAGAAGGAGGC	
PG0682	F: CGGTGAGTTCTATTATTGCG	123
	R: CAGCACCAGGCATGACCA	
PG1975	F: CGTGACGGGCATAAGACA	134
	R: AGTGAGTCGTGGGTTTAC	
PG1982	F: GTAATACCGAGGAAACTGAA	60
	R: GTGTTTCAGGGATAAGTCG	
PG2008	F: CTGCGGTTTCAACCAAGT	115
	R: ATACCGAACCTCGTCTAC	
PG0001	F: AGGTGGTCATGTTCCTCTCC	78
	R: TGACTACCCTCCTGCATTGG	

### Statistics

Significantly differentially expressed genes between the inoculated periodontitis and wild strains were identified using two class unpaired method in the Significant Analysis of Microarray software (SAM, version 3.02). Genes were determined to be significantly differentially expressed with a selection threshold of false discovery rate, FDR < 5 % and fold change > 2.0 in the SAM output result.

## Results

### Pathogenic effects of *P.gingivalis* W83 on rat oral cavity

**Fig. 1:**
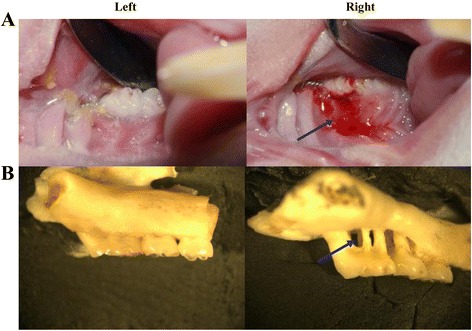
Pathogenic effects of *P.gingivalis* W83 on rat oral cavity. **(A)** The gingival tissues were inflammatory and bleeding after *P.gingivalis* W83 inoculation in rat oral cavity for 8 weeks. **(B)** Severe alveolar bone losses were found in rats with *P.gingivalis* W83 inoculation. Left: control groups. Right: rats inoculated with *P.gingivalis* W83 intraorally for 8 weeks

After *P.gingivalis* W83 inoculation in rat oral cavity for 8 weeks, the gingival tissues were inflammatory and bleeding (Fig. 1A). Severe alveolar bone losses were found in rats with *P.gingivalis* W83 inoculation (Fig. 1B). The distance between cementoenamel junction and alveolar bone crest (CEJ: ABC) was measured at proximal, middle and distal sites of buccal and palatal per molar, respectively. In first molars, second molars, third molars, the distances were significantly increased, which were 1216.00 ± 305.98 μm, 987.28 ± 238.14 μm, 725.11 ± 202.71 μm, compared with normal rats, which were 414.89 ± 209.67 μm, 300.44 ± 127.92 μm, 357.56 ± 281.06 μm.

### Identification of the inoculated *P.gingivalis* W83 in rat with periodontitis

**Fig. 2:**
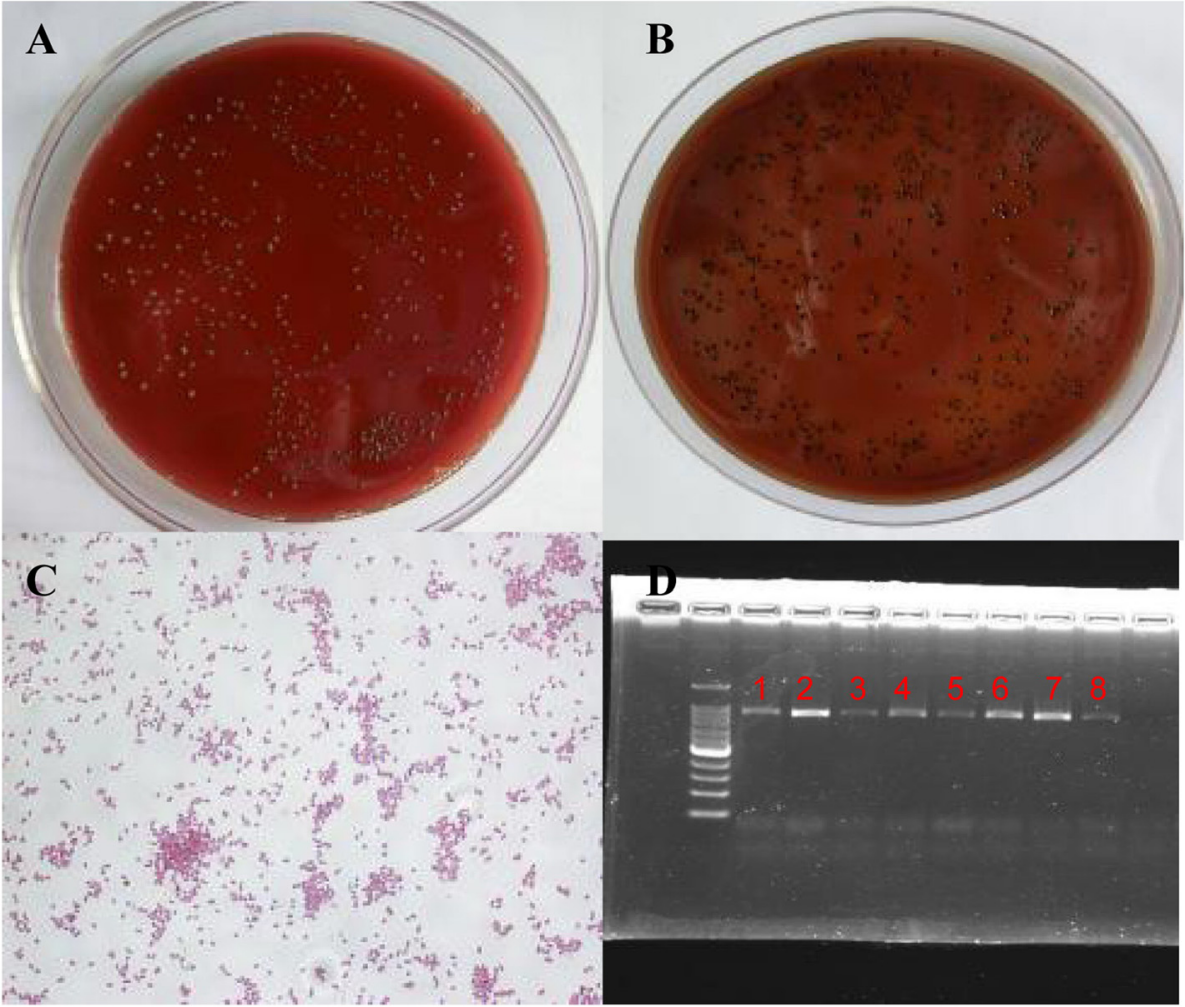
Isolation and identification of inoculated *P.gingivalis* W83. **(A)** Some suspicious black colonies were found from plaque mixture. **(B)** Suspious black colonies were pure cultured. **(C)** Gram staining proved the pure culture as gram-negative brevibacterium (×400). **(D)** Agarose gel electrophoresis proved that PCR fragment length was 857 bp. 1 and 2: Wild type *P.gingivalis* W83; 3–8: three inoculated *P.gingivalis* W83 samples for microarray analysis (two columns for each sample)

Suspicious gram-negative bacilli were taken from plaque culture. After pure subculture, gram staining and polymerase chain reaction (PCR) proved that the bacteria were *P.gingivalis* W83. PCR fragment length of the product was 857 bp, as shown in Fig. 2.

### Genes upregulated in the inoculated *P.gingivalis* W83

**Fig. 3:**
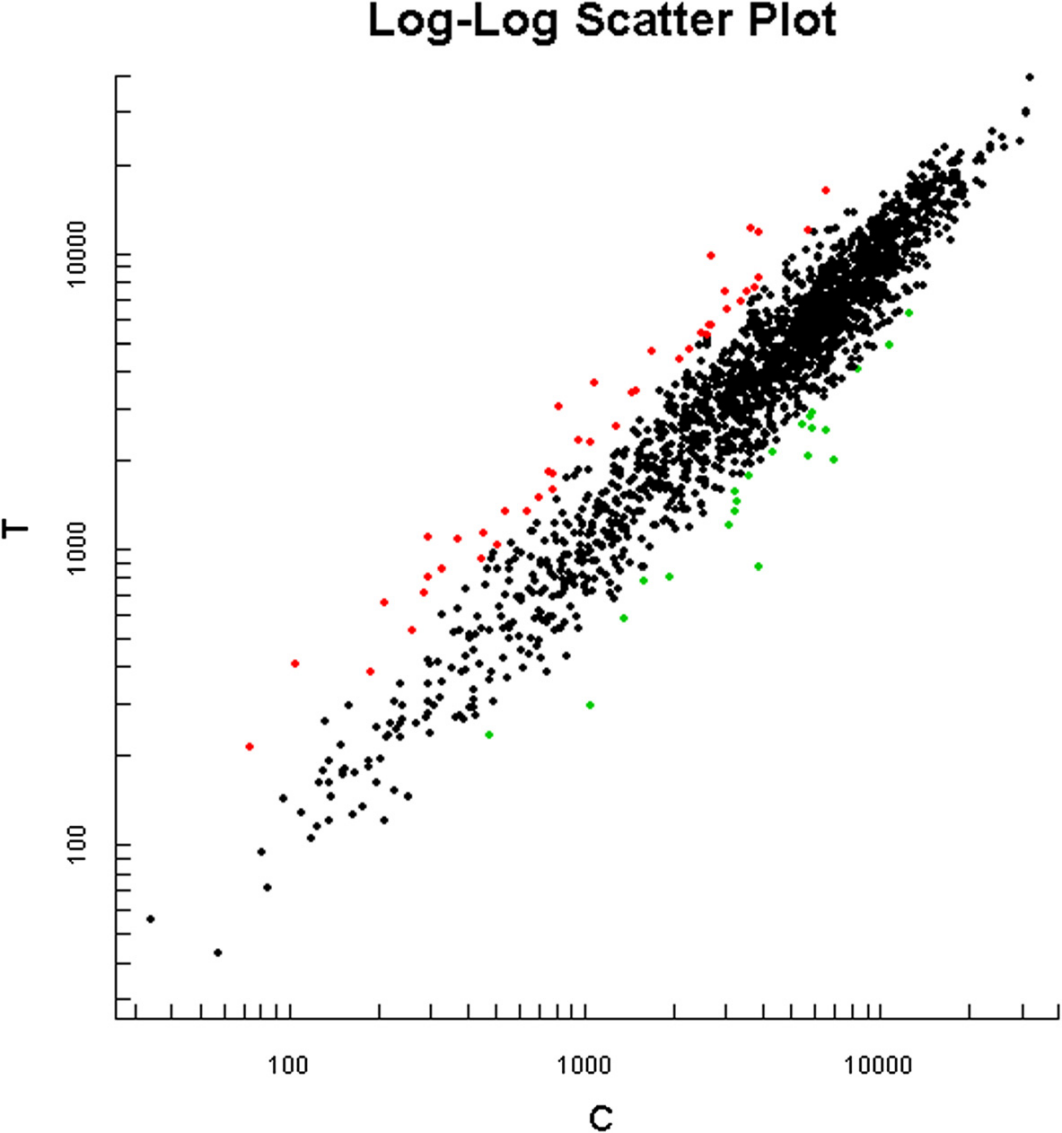
Genes analyzed by microarray in the inoculated *P.gingivalis* W83. Fluorescence signal strength values in X axes and Y axes represent control groups and experimental groups, respectively. Each data point was behalf of a gene chip hybridization signal. Red marking data points were T/C value ≥2, representing upregulated genes, and green marking data points were T/C value ≤0.5, representing downregulated genes

We determined the expression of 1786 genes by microarray analysis in *P.gingivalis* wild strain and *P.gingivalis* inoculated in oral cavity. The complete list of gene expression values has been deposited in NCBI’s Gene Expression Omnibus (http://www.ncbi.nlm.nih.gov/geo/query/acc.cgi?acc=GSE67608). The detection showed that 42 genes were up-regulated in the inoculated *P.gingivalis* W83 compared with wild strain (Table 2) (Fig. 3). In these upregulated genes, 30 expressed hypothetical proteins. Among other 12 genes, *PG0874*, *PG0009*, *PG0427*, *PG0942* and *PG0590* function as transposons. *PG0009*, *PG0427*, *PG0942* and *PG0590* encode ISPg5 transposases; and *PG0874* encodes an int protein as a mobilizable transposon.

**Table 2:** Genes upregulated in the inoculated P.gingivalis W83

Locus no.	Putative identification^α^	Cellular role^α^	Fold
PG0102	hypothetical protein		3.7076
PG2116	hypothetical protein		3.6850
PG1007	GntR family transcriptional regulator	Regulatory functions: DNA interactions	3.6786
PG0265	hypothetical protein		3.3714
PG1008	hypothetical protein		3.3338
PG1510	hypothetical protein		3.0890
PG1009	hypothetical protein		3.0258
PG1655	hypothetical protein		2.8935
PG0132	hypothetical protein		2.7298
PG2114	hypothetical protein		2.6986
PG2064	hypothetical protein		2.5952
PG1010	ABC transporter, ATP-binding protein	Transport and binding proteins: Unknown substrate	2.4885
PG0542	hypothetical protein		2.4794
PG1005	putative lipoprotein	Cell envelope	2.4686
PG1514	glycerol dehydrogenase-related protein	Unknown function: General	2.4537
PG0844	hypothetical protein		2.4324
PG0874	mobilizable transposon, int protein	Mobile and extrachromosomal element functions: Transposon functions	2.4307
PG0507	hypothetical protein		2.4290
PG1357	hypothetical protein		2.3151
PG1410	hypothetical protein		2.3094
PG0617	hypothetical protein		2.2814
PG0855	hypothetical protein		2.2153
PG0009	ISPg5, transposase Orf1	Mobile and extrachromosomal element functions: Transposon functions	2.1609
PG0427	ISPg5, transposase Orf1	Mobile and extrachromosomal element functions: Transposon functions	2.1431
PG0942	ISPg5, transposase Orf1	Mobile and extrachromosomal element functions: Transposon functions	2.1424
PG0541	hypothetical protein		2.1291
PG1398	hypothetical protein		2.1216
PG1027	hypothetical protein		2.1204
PG0590	ISPg5, transposase Orf1	Mobile and extrachromosomal element functions: Transposon functions	2.1151
PG1006	hypothetical protein		2.1122
PG0340	hypothetical protein		2.1119
PG0256	CvpA family protein	Unknown function: General	2.1090
PG0749	hypothetical protein		2.0834
PG1662	hypothetical protein		2.0788
PG2220	hypothetical protein		2.0771
PG1871	hypothetical protein		2.0589
PG2187	1,4-dihydroxy-2-naphthoate octaprenyltransferase	Biosynthesis of cofactors, prosthetic groups, and carriers: Menaquinone and ubiquinone	2.0471
PG0410	hypothetical protein		2.0409
PG1233	hypothetical protein		2.0367
PG0325	hypothetical protein		2.0349
PG0409	hypothetical protein		2.0259
PG1513	phosphoribosyltransferase, putative/phosphoglycerate mutase family protein	Energy metabolism: Other	2.0032

^α^Locus number, identification and functional classification according to JCVI *P.gingivalis* genome database

### Genes downregulated in the inoculated *P.gingivalis* W83

Compared with wild strain, 22 genes were down-regulated in the inoculated strain (Table 3) (Fig. 3). Among these genes, *PG0682*, *PG0683*, *PG0684*, *PG0282* and *PG0946* encode ABC transporters. Products of *PG0682*, *PG0683* and *PG0684* are putative permease proteins; and products of *PG0282* and *PG0946* are ATP-binding proteins. In addition, *PG2008* and *PG0283* also encode transport and binding proteins. All these proteins are involved in cell transmembrane transportation. Some downregulated genes encode proteins related to protein and nucleic acid metabolism, including *PG1129*, *PG1993*, *PG0001* and *PG0522*. Products of other genes are related to energy metabolism (*PG1042*), cell division (*PG0141*),bacterial pathogenicity (*PG1975*), and so on.

**Table 3:** Genes downregulated in the inoculated P.gingivalis W83

Locus no.	Putative identification^α^	Cellular role^α^	Fold
PG2008	TonB-dependent receptor, putative	Transport and binding proteins: Cations and iron carrying compounds	0.2234
PG0929	hypothetical protein		0.2806
PG1129	ribonucleotide reductase	Purines, pyrimidines, nucleosides, and nucleotides: 2'-Deoxyribonucleotide metabolism	0.2862
PG0684	ABC transporter, permease protein, putative	Transport and binding proteins: Unknown substrate	0.3594
PG0683	ABC transporter, permease protein, putative	Transport and binding proteins: Unknown substrate	0.3848
PG0682	ABC transporter, permease protein, putative	Transport and binding proteins: Unknown substrate	0.3938
PG0522	tRNA delta(2)-isopentenylpyrophosphate transferase	Protein synthesis: tRNA and rRNA base modification	0.4190
PG1648	RelA/SpoT family protein	Cellular processes: Adaptations to atypical conditions	0.4192
PG0282	ABC transporter, ATP-binding protein	Transport and binding proteins: Unknown substrate	0.4303
PG0946	ABC transporter, ATP-binding protein	Transport and binding proteins: Unknown substrate	0.4347
PG1042	glycogen synthase, putative	Energy metabolism: Biosynthesis and degradation of polysaccharides	0.4485
PG0890	alkaline phosphatase, putative	Central intermediary metabolism: Other	0.4552
PG1100	hypothetical protein		0.4813
PG1993	excinuclease ABC subunit C	DNA metabolism: DNA replication, recombination, and repair	0.4814
PG0226	transglutaminase-related protein	Unknown function: General	0.4823
PG0141	spoOJ protein	Cellular processes: Cell division	0.4865
PG0144	hypothetical protein		0.4888
PG1975	hemagglutinin protein HagC	Cellular processes: Pathogenesis	0.4941
PG1982	CRISPR-associated Cas1 family protein	Mobile and extrachromosomal element functions: Other	0.4950
PG1718	hypothetical protein		0.4955
PG0283	RND family efflux transporter MFP subunit	Transport and binding proteins: Unknown substrate	0.4965
PG0001	chromosomal replication initiation protein	DNA metabolism: DNA replication, recombination, and repair	0.4984

^α^Locus number, identification and functional classification according to JCVI *P.gingivalis* genome database

### Microarray result confirmation by real-time quantitative PCR

Among the upregulated and downregulated genes, we picked up 14 genes to detect the expression by real-time quantitative PCR. Consistent with microarray hybridization, real-time quantitative PCR detection showed similar expression trends in these genes (Table 4).

**Table 4:** Microarray result confirmation by real-time PCR

Gene	Fold increase measured by	
	Microarray analysis	Real-time PCR
PG1005	2.47↑	12.24 ± 2.12↑
PG1006	2.11↑	11.57 ± 1.06↑
PG1007	3.68↑	24.67 ± 3.67↑
PG1008	3.33↑	28.99 ± 4.56↑
PG1009	3.03↑	27.56 ± 2.66↑
PG1010	2.49↑	19.88 ± 3.41↑
PG0874	2.43↑	18.58 ± 2.08↑
PG1513	2.00↑	15.86 ± 2.12↑
PG0684	0.36↓	0.036 ± 0.004↓
PG0682	0.39↓	0.047 ± 0.005↓
PG1975	0.49↓	0.067 ± 0.011↓
PG1982	0.49↓	0.058 ± 0.007↓
PG2008	0.22↓	0.011 ± 0.002↓
PG0001	0.50↓	0.068 ± 0.008↓
16sRNA	-	1

## Discussion

Chronic periodontitis is initiated by periodontal pathogens, including *P.gingivalis*. Our study showed that *P.gingivalis* W83 induced rat gingival tissue inflammation, and alveolar bone loss, which is the key feature of periodontitis. Therefore, our study demonstrates that *P.gingivalis* W83 has pathogenic effects on rat oral cavity. After inoculation in rat oral cavity for 8 weeks, *P.gingivalis* W83 were isolated, and analyzed by microarray. In the detected 1786 genes, 42 genes were upregulated, whereas 22 genes were downregulated, indicating that the local periodontal environment can change the gene expression profile of *P.gingivalis* W83.

In the 42 upregulated genes, 30 expressed hypothetical proteins. Among other 12 genes, *PG0874*, *PG0009*, *PG0427*, *PG0942* and *PG0590* are in the same class in JCVI cell function classification. They all function as mobile extrachromosomal factor: transposon. Transposon is a removable genome DNA sequence, which can “jump” in genome from one location to another through the process of cutting and integration. Transposition is generally known to be triggered by cellular stress [12–14], therefore upregulation of these transposons suggests that *P.gingivalis* W83 inoculated in rat oral cavity may adapt local environment for its own survival, which is consistent with some other studies [15,16].

In the 22 downregulated genes, 7 genes encode transport and binding proteins. All these proteins are involved in cell transmembrane transportation. They can transport many substrates, such as metabolites, ion, sugar, amino acids, lipids, cholesterol and drugs [17]. *PG2008* encodes a TonB dependent receptor protein, responsible for iron transmembrane transportation [18]. As iron ion is necessary for the breeding and spreading of *P.gingivalis* W83, downregulation of *PG2008* suggests the subdued iron transferring and proliferation of *P.gingivalis* W83. There are 4 downregulated genes encoding proteins related to nucleic acid and protein metabolism. *PG1129* encodes a nucleotide reductase, which is related to purine, pyrimidine, nucleotide and DNA metabolism, and plays a regulating role in cell proliferation. Products of *PG1993* and *PG0001* are related to the metabolism of DNA, such as copy, restructuring and repair. Therefore, downregulation of these genes means that the proliferation of inoculated *P.gingivalis* W83 is in certain obstacles.

*PG1042* encodes a putative glycogen synthase, involved in biosynthesis and degradation of polysaccharides. Downregulation of *PG1042* suggests a disturbed energy metabolism. *PG0141* encodes a spoOJ protein related to cell division, and *PG1975* encodes hemagglutinin HagC related to pathogenicity of *P.gingivalis* W83. In addition, *PG1982* encodes a CRISPR protein related to CAS1 family. CRISPR/CAS system can protect bacteria against the encroachment by phage, and resist other chromosome genetic material and prevent from the expression of their genes [19–21]. Downregulation of *PG1982* suggests a decrease in the defense capability of *P.gingivalis* W83.

It should be noted that gene expression observed in this study was in mRNA level. As we have known, alterations in mRNA expression are not always consistent with those in protein expression. Therefore, observations in protein level of gene expression will be more convincing. However, it is impracticable to analyze the protein expression of all 64 genes with RNA expression alteration. Moreover, some products of these genes are still hypothetical proteins. Because the inoculated *P.gingivalis* was cultured outside the rat oral cavity for some days before RNA extraction, the RNA samples cannot exactly reflect the changes in gene expression after inoculation, although the results can still indicate which genes are upregulated or downregulated.

## Conclusions

Our study shows that *P.gingivalis* W83 has pathogenic effects on host, and local inflammatory oral environment alters the gene expression profile of *P.gingivalis* W83. Products of these upregulated and downregulated genes are mainly related to transposon functions, cell transmembrane transportation, protein and nucleic acid metabolism, energy metabolism, cell division and bacterial pathogenicity. These changes may lead to decreased proliferation and pathogenicity of *P.gingivalis* W83, and favor themselves to adapt local environment for survival.

## Abbreviations

P.gingivalis: Porphyromonas gingivalis
PCR: Polymerase chain reaction
SPF: Specific pathogen free
ATCC: American Type Culture Collection
CFU: Colony forming unit
CEJ: Cementoenamel junction
ABC: Alveolar bone crest
CRISPR: Clustered Regularly Interspaced Short Palindromic Repeats
CAS: CRISPR associated genes

## References

[1] Williams RC (1990). Periodontal disease. N Engl J Med.

[2] Aruni AW, Zhang K, Dou Y, Fletcher H (2014). Proteome analysis of coinfection of epithelial cells with Filifactor alocis and Porphyromonas gingivalis shows modulation of pathogen and host regulatory pathways. Infect Immun.

[3] Nissen L, Sgorbati B, Biavati B, Belibasakis GN (2014). Lactobacillus salivarius and L. gasseri down-regulate Aggregatibacter actinomycetemcomitans exotoxins expression. Ann Microbiol.

[4] Kerr JE, Abramian JR, Dao DH, Rigney TW, Fritz J, Pham T (2014). Genetic exchange of fimbrial alleles exemplifies the adaptive virulence strategy of Porphyromonas gingivalis. PLoS One.

[5] Baker PJ (2000). The role of immune responses in bone loss during periodontal disease. Microbes Infect.

[6] Anaya-Bergman C, Rosato A, Lewis JP (2015). Iron- and hemin-dependent gene expression of Porphyromonas gingivalis. Mol Oral Microbiol.

[7] Phillips P, Progulske-Fox A, Grieshaber S, Grieshaber N (2014). Expression of Porphyromonas gingivalis small RNA in response to hemin availability identified using microarray and RNA-seq analysis. FEMS Microbiol Lett.

[8] Moon JH, Lee JH, Lee JY (2014). Microarray analysis of the transcriptional responses of Porphyromonas gingivalis to polyphosphate. BMC Microbiol.

[9] Azelmat J, Larente JF, Grenier D (2015). The anthraquinone rhein exhibits synergistic antibacterial activity in association with metronidazole or natural compounds and attenuates virulence gene expression in Porphyromonas gingivalis. Arch Oral Biol.

[10] Yoshimura M, Ohara N, Kondo Y, Shoji M, Okano S, Nakano Y (2008). Proteome analysis of Porphyromonas gingivalis cells placed in a subcutaneous chamber of mice. Oral Microbiol Immunol.

[11] Baker PJ, Dixon M, Roopenian DC (2000). Genetic control of susceptibility to Porphyromonas gingivalis-induced alveolar bone loss in mice. Infect Immun.

[12] Zhang Z, Saier MH (2011). Transposon-mediated adaptive and directed mutations and their potential evolutionary benefits. J Mol Microbiol Biotechnol.

[13] Wheeler BS (2013). Small RNAs, big impact: small RNA pathways in transposon control and their effect on the host stress response. Chromosome Res.

[14] Arnault C, Dufournel I (1994). Genome and stresses: reactions against aggressions, behavior of transposable elements. Genetica.

[15] Hendrickson EL, Xia Q, Wang T, Lamont RJ, Hackett M (2009). Pathway analysis for intracellular Porphyromonas gingivalis using a strain ATCC 33277 specific database. BMC Microbiol.

[16] Xia Q, Wang T, Taub F, Park Y, Capestany CA, Lamont RJ (2007). Quantitative proteomics of intracellular Porphyromonas gingivalis. Proteomics.

[17] Park Y, Yilmaz O, Jung IY, Lamont RJ (2004). Identification of Porphyromonas gingivalis genes specifically expressed in human gingival epithelial cells by using differential display reverse transcription-PCR. Infect Immun.

[18] Létoffé S, Delepelaire P, Wandersman C (2004). Free and hemophore-bound heme acquisitions through the outer membrane receptor HasR have different requirements for the TonB-ExbB-ExbD complex. J Bacteriol.

[19] Pourcel C, Salvignol G, Vergnaud G (2005). CRISPR elements in Yersinia pestis acquire new repeats by preferential uptake of bacteriophage DNA, and provide additional tools for evolutionary studies. Microbiology.

[20] Haft DH, Selengut J, Mongodin EF, Nelson KE (2005). A guild of 45 CRISPR-associated (Cas) protein families and multiple CRISPR/Cas subtypes exist in prokaryotic genomes. PLoS Comput Biol.

[21] Grissa I, Vergnaud G, Pourcel C (2007). The CRISPRdb database and tools to display CRISPRs and to generate dictionaries of spacers and repeats. BMC Bioinformatics.

